# Patterns of National Cancer Institute‐Sponsored Clinical Trial Enrollment in Black Adolescents and Young Adults

**DOI:** 10.1002/cam4.4292

**Published:** 2021-09-30

**Authors:** Michael Roth, Melissa Beauchemin, Justine M. Kahn, Archie Bleyer

**Affiliations:** ^1^ Division of Pediatrics Department of Pediatrics University of Texas MD Anderson Cancer Center Houston Texas USA; ^2^ Herbert Irving Comprehensive Cancer Center Columbia University Irving Medical Center New York NY USA; ^3^ Knight Cancer Institute and Department of Radiation Medicine Oregon Health and Science University Portland Oregon USA

**Keywords:** adolescents and young adults, Black or African American, cancer treatment trials, disparities

## Abstract

**Background:**

Both adolescent and young adult (AYA) and Black or African American (hereafter referred to as Black) cancer patients are historically under‐enrolled in cancer treatment trials (CTT). The purpose of this study was to quantify enrollment of Black AYAs in National Cancer Institute (NCI)‐sponsored CTTs overall and by age, sex, and cancer diagnosis during 2000–2015.

**Methods:**

Utilizing data from NCI’s Cancer Therapy Evaluation Program and the Surveillance, Epidemiology and End Results (SEER) Program, we assessed CTT enrollment in Black patients with cancer and measured changes in enrollment over time between the study periods 2000–2007 and 2008–2015. Enrollment patterns were compared across age groups (≤14 years [y], 15–19y, 20–29y, 30–39y and 40+ years), sex, and cancer diagnosis.

**Results:**

From 2000 through 2015, <3% of Black AYAs (20–39y) enrolled on CTTs. While AYAs had significantly higher cancer incidence than children, 20.5% fewer Black AYAs enrolled on CTTs. Enrollment was lowest among Black males 20–29y, with a mean of 18 enrolling in CTTs annually. The proportion of AYA enrollees who were Black did not change significantly over time periods (2000–2007 vs 2008–2015).

**Conclusions:**

Few Black AYAs enroll in CTTs each year. Given known benefits of clinical trial participation and the well‐documented racial and age‐related differences in cancer outcomes, addressing barriers to enrollment in these patients may, in turn, reduce disparities. Targeted interventions aimed at increasing the CTT enrollment of Black cancer patients, particularly young Black men, are urgently needed.

**Precis:**

This study documents that compared with Black children, Black adolescent, and young adult (AYA) patients were less likely to enroll in NCI‐sponsored CTTs from 2000 to 2015. Black AYA male enrollment decreased with increasing age, highlighting disparities among this specific population in CTT enrollment.

## Graphical abstract

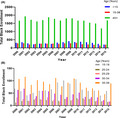

## 1 INTRODUCTION

The National Cancer Institute (NCI) defines adolescents and young adults (AYA; age 15–39 years [y]) as a vulnerable population because they have not experienced the same survival gains as other age groups over the last three decades and because cancer remains a leading cause of death among these patients.^1^ Recent efforts to explain these disparities have focused on age‐related differences in access to high‐quality healthcare, the unique psychosocial needs of AYAs,^2^ and the distinct biology of many cancers common among AYAs.^3^ An area of particular importance is the consistent under‐enrollment of AYAs on cancer treatment trials (CTTs). Proposed hypotheses for the AYA gap in clinical trial enrollment include higher likelihood of receiving care at community rather than academic centers^4^; the lack of CTTs available to AYAs; and inconsistent referral patterns to centers with available CTTs.^5^, ^6^ As survival outcomes remain stagnant for many AYA cancer subtypes,^7^ more research is needed to understand the underlying causes of the “AYA gap” in CTT enrollment.^8^, ^9^, ^10^, ^11^

Equally pressing is the under‐enrollment of Black or African American (hereafter referred to as Black) patients in CTTs. Black patients with cancer have worse outcomes than their White and Hispanic counterparts across almost every diagnosis.^12^ They are also less likely to enroll on CTTs for reasons that are not well understood.^13^, ^14^, ^15^ In 2019, approximately 200,000 new cancer cases and 73,000 cancer deaths were reported among Black individuals in the United States (U.S.).^12^ Federal efforts designed to bring CTTs to community‐based and minority medical practices, including the National Institutes of Health (NIH) Revitalization Act, (which mandates the inclusion of minority groups in NIH‐funded research), the NCI Community Clinical Oncology Program, and the NCI Community Oncology Research Program have contributed to improvements in minority CTT enrollment; however, Black patients still remain under‐represented in CTTs.^16^, ^17^, ^18^, ^19^

The benefits and importance of CTT enrollment are multiple: They provide participants access to novel therapies, facilitate biospecimen collection for basic and translational research, enable studies of psychosocial and supportive care needs, and support research on quality of life and other non‐survival outcomes.^4^, ^20^, ^21^ As all of these factors are essential to improving cancer care across populations, under‐enrollment of AYAs and Black patients both restricts patient access to treatment innovations and limits the field's ability to determine best practices for different age or racial/ethnic groups.

Despite CTT enrollment gaps in both AYAs and Black patients with cancer, there is a paucity of data specifically regarding the representation of Black AYAs on CTTs. We hypothesize that there is some overlap in the known barriers to CTT enrollment among both Black patients and AYAs with cancer, but there may also be barriers that are unique or heightened in this population. To begin to address this gap in the literature, we identified a population‐based cohort of Black children, AYAs and older adults diagnosed with cancer in the U.S. between 2000 and 2015 and: (1) quantified enrollment of patients on CTTs, focusing on AYAs; (2) compared enrollment rates across children, AYAs, and older adults (pediatric: ≤14 years [y], AYA: 15–39y, older adult: 40+), and within the AYA age group alone; and (3) examined the influence of sex and cancer diagnosis on CTT in the AYA cohort.

## 2 METHODS

### 2.1 Data sources

Data on patients enrolled in NCI‐sponsored CTTs from 2000 through 2015 were requested and obtained from the Cancer Therapy Evaluation Program (CTEP) database. The data were deidentified prior to transfer, exported in an Excel file (Microsoft) and included patient age, sex, diagnosis, race, and year of trial enrollment. All patients included in the database consented and enrolled on Institutional Review Board (IRB) approved NCI‐sponsored studies. Additional IRB approval was not sought for this analysis as all data were deidentified and there was no possibility of linking enrollment data to individual patients. The annual cancer incidences for Black patients ages 15–19y, 20–24y, 25–29y, 30–34y, and 35–39y from 2000 through 2015 were obtained from the Surveillance, Epidemiology, and End Results (SEER) Program database.^22^ SEER is a population‐based, comprehensive de‐identified database of annual cancer incidence and mortality in the U.S. Intercensal estimates from the U.S. Census Bureau were utilized to determine the total number of Black AYAs living in the U.S. during the study years.^23^, ^24^

### 2.2 Statistical analysis

We calculated the total number of children, AYAs, and older adults of any race or ethnicity enrolled in CTTs annually from 2000 to 2015 (16 years total). Within this population, we then focused on the numbers of Black patients for the duration of the study. Black CTT enrollees were split into three age groups: children (≤14y), AYAs (15–39y), and older adults (age ≥40 years). The number of Black AYAs enrolled in CTTs each year within the AYAs only (15–19y, 20–24y, 25–29y, 30–34y, and 35–39y) was also calculated. Patients whose race was unknown or reported as mixed race were excluded from the analysis.

Among the Black patients diagnosed within the study years, we compared the proportion of AYA enrollees with the proportions of children and adult enrollees using the Fisher exact test and the χ^2^ test. The χ^2^ test was also used to compare proportions of enrollees ages 15–19y, 20–29y, and 30–39y. The proportion of AYA enrollees across age groups was also assessed by sex and cancer type and SEER incidence rates and U.S. Census Bureau data were used to determine the proportion of the population with each cancer that is Black by age and sex. Finally, the χ^2^ test was used to compare the proportions of children, AYAs, and older adults enrolled in CTTs during 2000–2007 with the proportions enrolled during 2008–2015.

Annual SEER estimated enrollment proportions were calculated by first estimating the total number of Black AYAs diagnosed with cancer each year in 5‐year age intervals. The SEER crude incidence rate (new cases/100,000 people) for each age group was multiplied by the number of Black individuals estimated to be living in the U.S. in the corresponding year and then divided by 100,000. To obtain the estimated enrollment proportion, the number of Black AYA enrollments was divided by the estimated number of Black AYAs diagnosed with cancer each year in 5‐year age intervals. For all tests, *p*‐values <0.05 were considered statistically significant. All analyses were conducted using GraphPad Prism 8 (GraphPad Software Inc.).

## 3 RESULTS

### 3.1 Black AYAs comprise a small proportion of CTT enrollees

**FIGURE 1:**
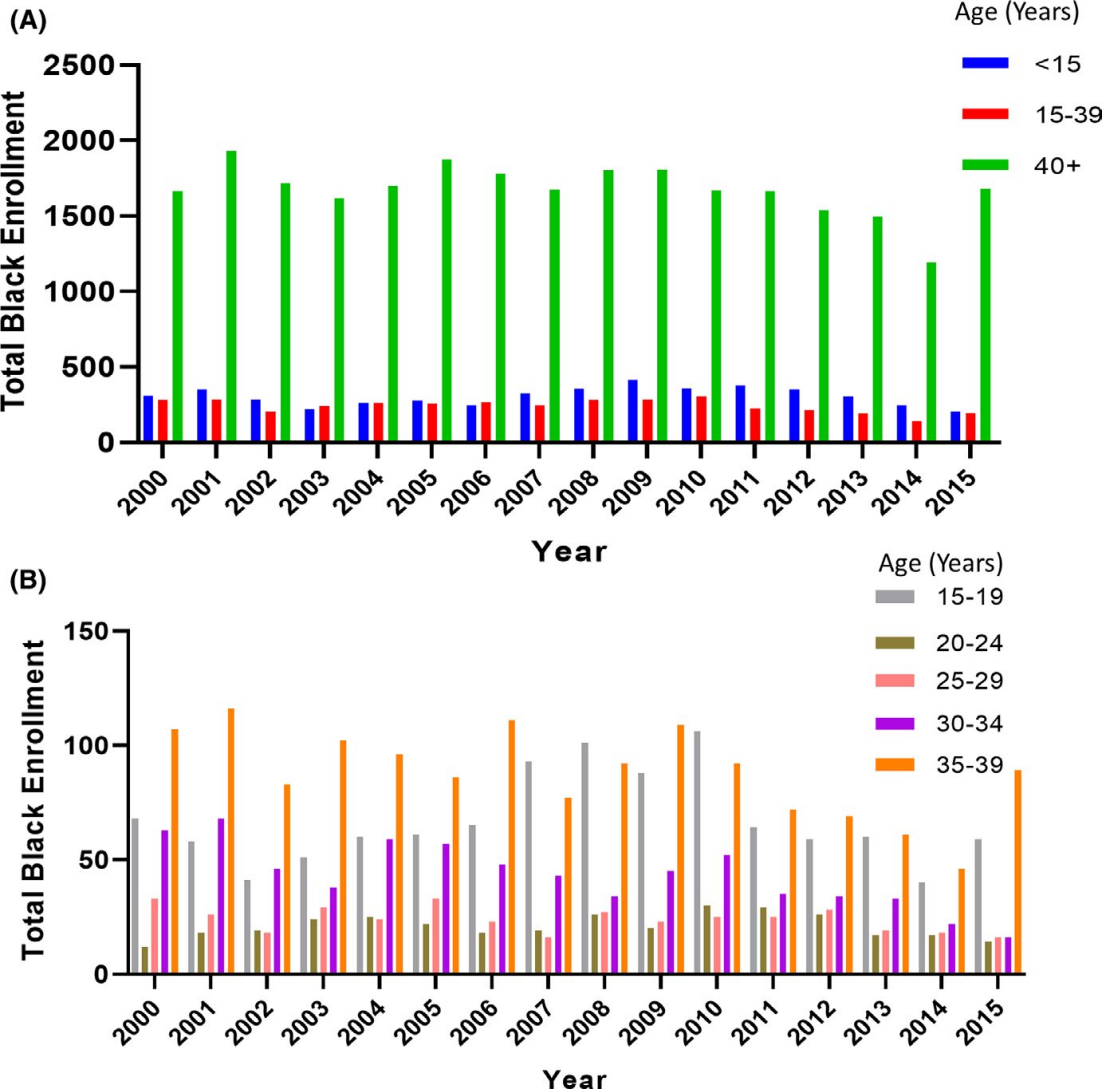
Total numbers of Black patients enrolled in NCI‐sponsored CTTs, 2000–2015. (A) Total numbers of Black children (age <15 years), Black AYAs (age 15–39 years), and Black adults (age 40+ years) enrolled in CTTs. (B) Total numbers of Black AYAs enrolled in CTTs, stratified by age.

From 2000 through 2015, NCI‐sponsored CTTs enrolled 46,120 children, 34,348 AYAs, and 334,296 older adults with a single reported race. Of these total enrolled patients, 35,600 were Black: 4897 (10.6%) were children, 3893 (11.3%) were AYAs, and 26,810 (8.0%) were older adults. Annual enrollment numbers in each group are shown in Figure 1A. On average, 306 children, 243 AYAs, and 1675 older adults have enrolled on CTTs annually. Black AYAs had >4 times higher cancer incidence compared with children (59.5 vs 12.7 per 100,000, respectively); however, 1004 more children than AYAs enrolled on CTTs during these years, a difference of 20.5%.^22^

The annual CTT enrollment numbers by age group within the AYA‐aged patients only are shown in Figure 1B. The enrollments were highest for those ages 15–19 years (N = 1074) and 35–39 years (N = 1408). Compared with AYAs ages 20–24y (N = 336) and 25–29y (N = 383), those ages 15–19y were 3.2 times and 2.8 times higher, respectively.

**FIGURE 2:**
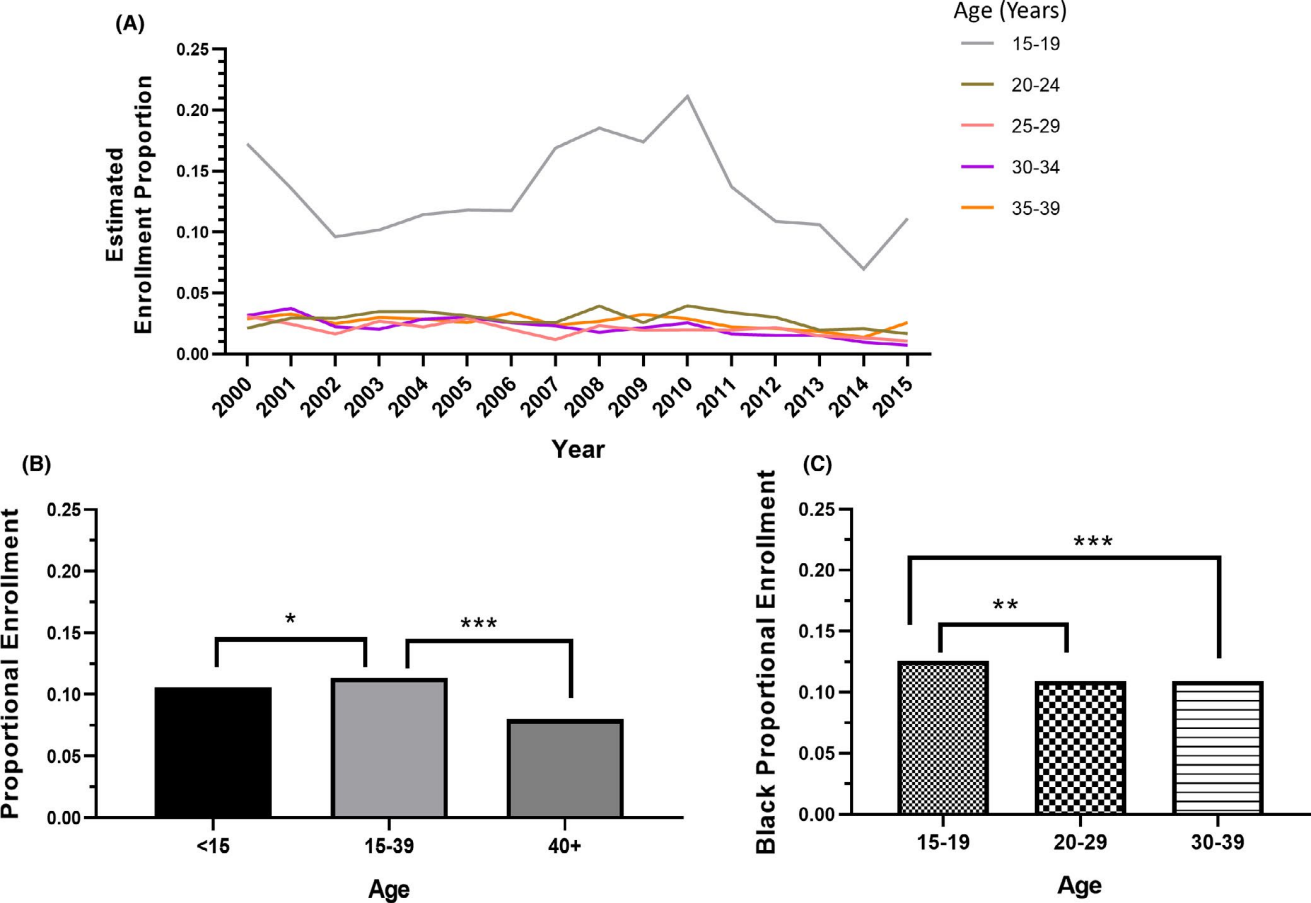
Proportions of NCI‐sponsored CTT enrollees who were Black, 2000–2015. (A) Proportions of Black AYA enrollees, stratified by age, as estimated with SEER Program data. (B) Average proportions of Black pediatric (age <15 years), AYA (age 15–39 years), and older adult (age 40+ years) enrollees. (C) Average proportions of AYA enrollees who were Black, stratified by AYA age group. *p<0.05, **p<0.001, ***p<0.001.

SEER estimated rates for Black patients were less than 3% in each AYA specific age group except patients aged 15–19y. Estimated CTT enrollment rates were highest for Black patients ages 15–19y (13.2%) and lowest for ages 25–29y (2.0%) (Figure 2A).

During the study period, 20.3%, 34.0%, and 45.7% of the U.S. population were under 15 years of age, between age 15 and 39, and older than 39 years, respectively. Of individuals younger than age 15, patients age 15–39 and patients older than 39, 15.1%, 14.5% and 11.1% were Black, respectively.^23^, ^24^ In 2010, cancer incidence in the U.S. population was 474.7 per 100,000 and was similar in Black individuals compared with White individuals (485.9 vs 486.3, respectively). Cancer incidence increased with increasing age. Black children and AYAs had a lower cancer incidence compared with White children and AYAs; however, Black adults age 50–69 years had a higher cancer incidence compared with White adults.^22^

### 3.2 Proportions of enrolled patients differed across Black children, AYAs, and older adults

The proportion of AYAs who enrolled in CTTs who were Black (11.3%) was significantly higher than the proportion of children who were Black (10.6%; *p *= 0.04) and of older adults (8.0%; *p *< 0.001) (Figure 2B). Among AYA enrollees, the proportion of Black patients age 15–19 years (12.6%) was significantly higher than that of those aged 20–29y (10.9%; *p *= 0.002) or that of those aged 30–39 years (10.9%; *p *< 0.001) (Figure 2C).

### 3.3 AYA enrollment remained stagnant from 2000 through 2015

**FIGURE 3:**
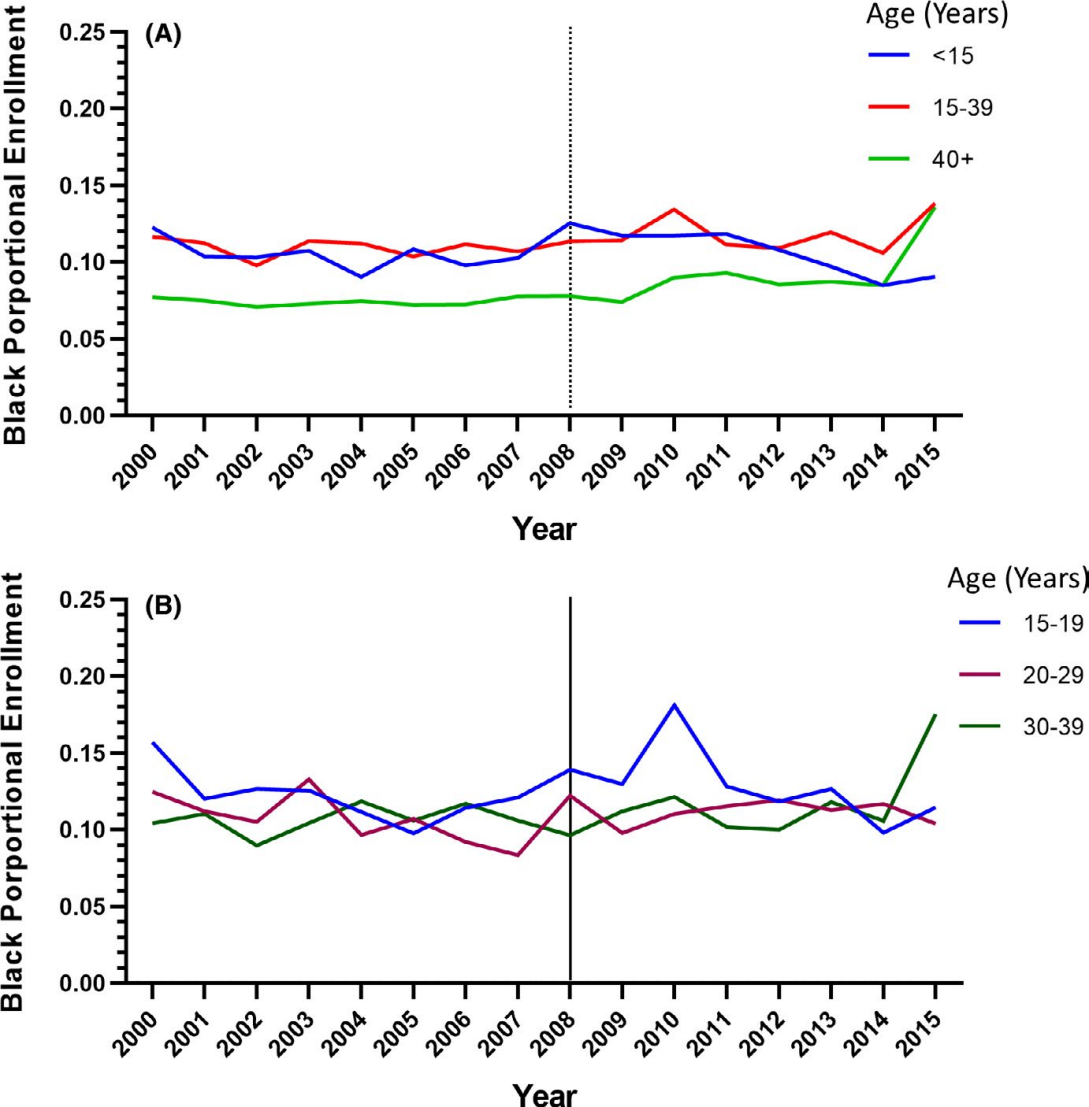
Change in proportion of NCI‐sponsored CTTs enrollees who were Black, 2000–2015. (A) Change in proportion of pediatric (age <15 years), AYA (age 15–39 years), and older adult (age 40+ years) enrollees who were Black over time. (B) Change in proportions of AYA enrollees who were Black, stratified by age group, over time.

The proportion of Black AYAs who enrolled on CTTs did not increase between 2000 and 2015 (Figure 2A) and did not significantly differ between 2000–2007 and 2008–2015 (10.9% vs 11.8%, *p *= 0.33) (Figure 3A). In addition, the proportions of Black AYA enrollees did not change significantly across age groups for the two study periods: 15–19 years (12.0% vs 13.2%; *p *= 0.15), 20–29 years (10.6% vs 11.2%; *p *= 0.54), or 30–39 years (10.7% vs 11.2%; *p *= 0.21) (Figure 3B).

### 3.4 Among Black AYAs, CTT enrollment rates differed by age, sex, and tumor type

**FIGURE 4:**
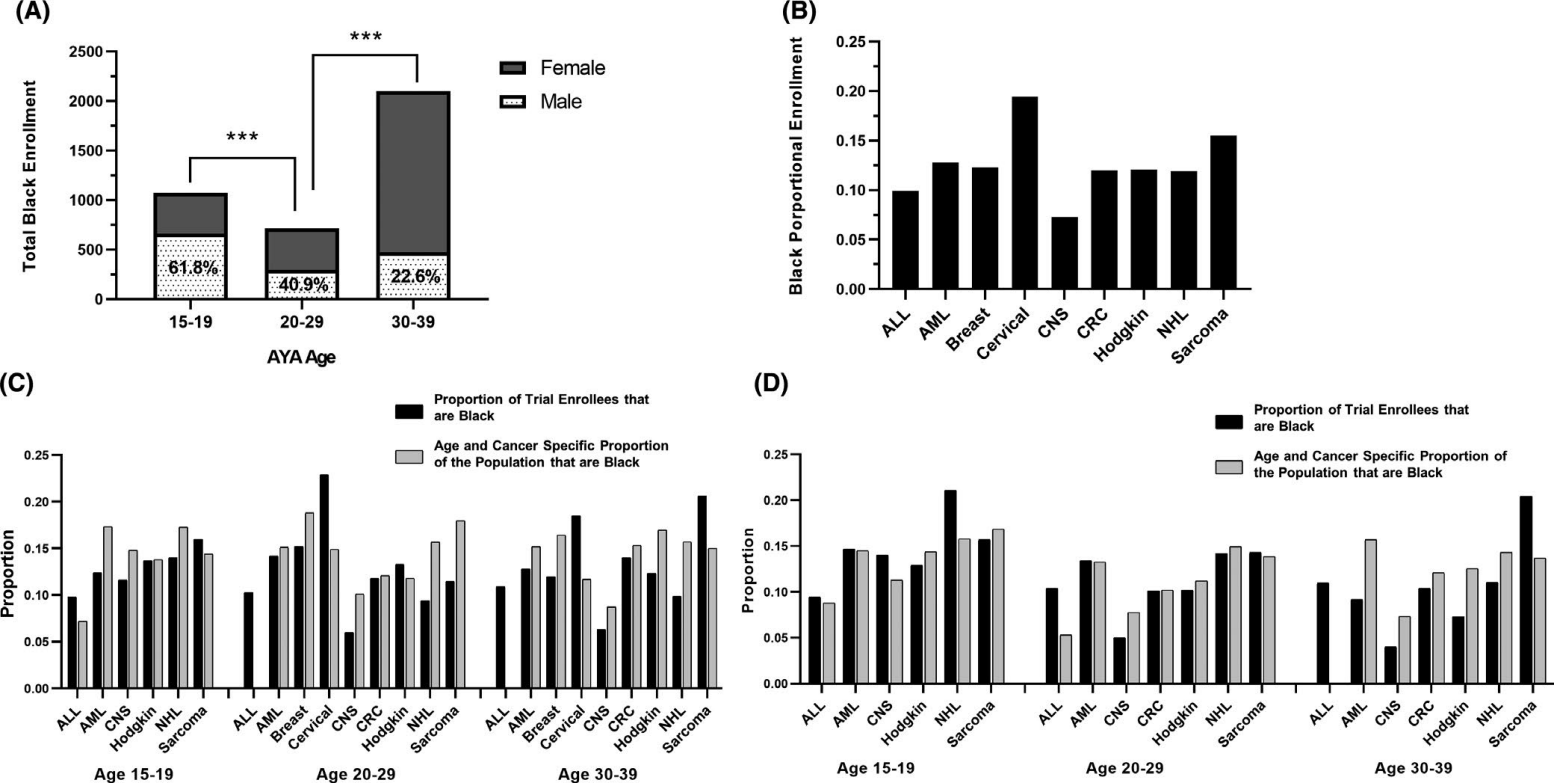
Black AYAs’ rates of CTT enrollment differ by sex and cancer type. (A) Total numbers of Black AYAs enrolled in CTTs, stratified by sex and age, 2000–2015. ***p<0.001. (B) Proportions of Black AYAs enrolled in CTTs, stratified by cancer type. ALL, acute lymphoblastic leukemia; AML, acute myeloid leukemia; CNS, central nervous system; CRC, colorectal cancer; NHL, non‐Hodgkin lymphoma. (C) Proportion of female AYA trial enrollees that were Black by age and cancer diagnosis. The light gray bar represents the proportion of females in the U.S. cancer population that were Black by age and cancer diagnosis. Data was not available on the incidence of ALL in female AYAs age 20–29 and 30–39. (D) Proportion of male AYA trial enrollees that were Black by age and cancer diagnosis. The light gray bar represents the proportion of females in the U.S. cancer population that were Black by age and cancer diagnosis.

The proportion of AYA enrollees who were male decreased with increasing age. Male AYAs represented 61.8%, 40.9%, and 22.6% of enrollees ages 15–19y, 20–29y, and 30–39y, respectively (Figure 4A). On average, 42, 18, and 30 Black men of ages 15–19y, 20–29y, and 30–39y, respectively, enrolled in CTTs annually. The proportion of AYA enrollees who were Black differed by cancer diagnosis and was highest among patients with cervical cancer (19.4%) and lowest among patients with central nervous system tumors (7.3%) (Figure 4B). The proportion of AYA enrollees who were Black compared with the proportion of cancer patients who were Black in the U.S. population varied by sex, age, and cancer diagnosis (Figure 4C,D).

## 4 DISCUSSION

In this study, we found that among Black patients diagnosed with cancer between 2000 and 2015, AYAs were less likely than children to enroll on NCI‐sponsored CTTs. Furthermore, the proportion of AYA CTT enrollees who were Black did not increase during the 16 years of the study period, despite national efforts to expand access to vulnerable populations including AYAs and minority patients.^18^ Most Black AYA enrollees were women ages 30–39y, with very few Black men ages 20–29y enrolling on trials. Overall, our findings highlight an urgent need to understand barriers to and address the under‐enrollment of Black AYAs in CTTs, particularly men between 20 and 29 years.

It is established that CTTs are the most effective way to study cancer biology and treatment outcomes and that enrolling in prospective trials improves both survival and health‐related quality of life.^11^ It is also established that both AYAs and Black patients with cancer in the U.S. continue to lag behind on the survival curves compared to children and non‐minority patients. Thus, improving the CTT enrollment rates of Black AYAs’ could address overarching concerns of health disparities, racial inequities, and access to cancer care barriers among these high‐risk populations. As only 3%–14% of AYAs with cancer enroll in CTTs, low participation rates in these aged patients are very likely to be implicated in disparate outcomes.^25^, ^26^, ^27^, ^28^ Moreover, Black AYA CTT enrollment rates are consistently lower than overall rates,^19^ limiting the ability to study optimal therapeutic and supportive care approaches in this population.

Similar to our findings, a prior study of NCI‐sponsored CTT enrollments found that minority enrollments decreased with increasing age.^29^ The current study builds on this by examining CTT enrollment for narrower age groups of Black AYAs to determine the extent to which age, sex and tumor type impact CTT enrollment. Black AYA enrollment was found to be 11.3% among all AYA CTT enrollees, which is lower than the proportion of Black AYAs in the U.S. (>13%). Perhaps even more concerning is how few Black AYAs with cancer enrolled on trials (SEER estimate <3%). This may be related to a combination or compounding of CTT enrollment barriers that impact both AYAs and Black patients; however, further research is needed to explain this finding.

Our study findings are important in the context of the well‐described survival gap between Black and White AYAs across several cancers.^13^, ^30^ The cause of these disparities is likely multi‐factorial and includes system‐level (access to high‐quality care, availability of clinical trials), provider‐level (workflow challenges, lack of awareness of guideline‐based care of available clinical trial), and patient‐level (financial toxicity, mistrust of the medical system).^14^, ^31^ Minority populations in the U.S. are more likely than White patients to have public or no insurance, are more likely to receive treatment at under‐resourced hospital settings, and are more likely to present with advanced stage disease.^32^, ^33^, ^34^, ^35^ Similarly, AYAs are likely to be under‐insured and this, in conjunction with socioeconomic status and geographic factors may contribute to their treatment locations, which in turn, likely impacts likelihood of CTT enrollment.^25^, ^36^ Further, health insurance status and socioeconomic status (SES) are both significantly associated with survival in AYAs with cancer, suggesting differences in the type and quality of care low‐income patients may be receiving across the U.S.^37^, ^38^, ^39^ Numerous studies have shown that treatment at large academic centers is associated with improved survival rates for AYAs with cancer. One possible factor contributing to these findings related to location‐of‐care may be a difference in clinical trial enrollment rates across community and academic centers.^11^ National efforts, such as the NCI Community Oncology Research Program that aims to bring cancer clinical trials to people in their communities to increase representation on clinical trials and ultimately reduce disparities, should continue efforts toward understanding and addressing the multilevel barriers to CTT enrollment. Our study findings further support the urgent need for these efforts, specifically toward raising awareness about and addressing challenges in accessing clinical trials for Black AYA communities.

We found that more children (age ≤14y) than AYAs (15–39y) enrolled in CTTs during the study period. This is a concerning finding, as the cancer incidence among AYAs is more than 7 times the incidence among children. We were unable to account for number of clinical trials open for AYA enrollment during this time period so further work is needed.^40^, ^41^ Lower Black AYA and overall enrollment in 2014, when the NCI Clinical Trials Network was restructured, may have been secondary to a decrease in the overall number of available trials. As expected, we identified differences in CTT enrollment among the AYAs with those 15–19y enrolling more often than those 20–39y. Adjusting for location‐of‐care will be important in future analyses. This may be due to differences in treatment settings for younger AYAs compared with older AYAs, as patients age 15–18 years are frequently treated by pediatric cancer programs at NCI‐supported centers, whereas older AYAs are more likely to be treated at community treatment sites.^25^, ^42^, ^43^ Further work is needed to clarify the reasons for this finding; however, it suggests that increasing access to clinical trials for Black AYAs, such as through collaboration between academic and community providers may be a potential strategy to increase CTT enrollment in Black AYAs.

The results of our study highlight differences in Black AYA enrollment in CTTs by both sex and cancer type. Among Black women, CTT enrollment was significantly higher among individuals ages 30–39y than in those ages 20–29y, which may be a result of the higher incidence of cervical cancer and breast cancer with increasing age. Our finding that more AYA women than men enrolled in CTTs may reflect a greater burden of gynecologic and breast cancers in young adult women, which has been a large focus of cooperative group efforts in recent decades.^44^ Other factors, including mistrust of the medical establishment and research due to historical racial injustice in clinical trials, may contribute to Black AYAs, and particularly Black men's relatively low enrollment in CTTs.^45^ Similarly, the possibility of discordant attitudes^46^ or implicit bias between provider and patient needs to be considered when determining what factors, either patient, provider or both, may contribute to low enrollment among Black AYA men.

Interestingly, the proportion of sarcoma CTT enrollees who were Black was higher than most of the other cancers, despite the fact the <3% of all patients with Ewing sarcoma are Black.^47^ If Ewing sarcoma patients are removed from the analysis, the proportion of enrollees with sarcoma who are Black is >20%. This may be a reflection of the low mean age of sarcoma patients enrolled on trials (19.7y); however, this does not completely explain the finding. The Children's Oncology Group (COG) conducts many of the sarcoma trials within the NCI’s National Clinical Trials Network and thus many, if not most, of these patients were likely enrolled at academic pediatric oncology sites. If this is the case, it further supports the hypothesis that these sites, with the resources and infrastructure to run clinical trials, are well‐equipped to enroll patients onto trials. However, a high proportion of AYA enrollees with sarcoma in their 30s were Black, and it is unlikely these patients were treated at COG sites, suggesting other factors may support Black AYA sarcoma patient enrollment on trials.

The proportion of AYAs enrolled on NCI‐supported trials who were Black mirrored the overall proportion of AYAs with cancer who were Black in the population for many cancers (Figure 4), and for a few cancer types, it appears that Black AYAs may have been over‐represented, comparatively. It is important to note that the denominator, the total number of AYAs enrolling on NCI trials is exceedingly low and, while Black AYAs may enroll at similar rates compared to non‐Black AYAs, rates of enrollment are dismal in both patient populations. The minimal total number of Black AYAs enrolling on cancer clinical trials each year does not allow for studies to further identify and address disparities in outcomes for this population due to limited study power.

An important limitation of this study was the inability to identify the true proportion of Black AYAs enrolling in NCI‐supported CTT. The study did not include fluctuations in the number and types of trials that were available for AYA enrollment over the study period, which could have impacted the absolute numbers of enrolled patients. However, our reporting of data on Black AYAs’ enrollment in CTTs by participant characteristics (age, sex, cancer type) provides both important and generalizable data to inform follow‐up analyses examining multilevel barriers to enrollment for future intervention. The analysis was also not able to capture the location‐of‐care or whether there were geographic barriers to enrollment or treatment at an NCI cancer center.

We found that compared to Black children with cancer, Black AYAs, and particularly male Black AYAs under‐represented on CTT in the US. Future research is needed to both better characterize the actual representation of AYAs on clinical trials, specifically Black AYAs and other under‐represented populations, and to systematically identify the socioeconomic, sociocultural, institutional, and systemic barriers that influence the enrollment of AYAs, specifically Black AYAs onto CTTs. Only then can these barriers be effectively addressed at the individual patient and institution level, but importantly through broader health policy reform that optimizes access for and enrollment of under‐represented AYAs into CTTs in the US.

## 5 DISCLAIMER

The opinions expressed by the authors are their own and this material should not be interpreted as representing the official viewpoint of the Children's Oncology Group, the National Institutes of Health, or the National Cancer Institute.

## 6 ETHICS STATEMENT

All patients included in the database utilized in this analysis consented and enrolled on Institutional Review Board IRB) approved NCI‐sponsored studies. Additional IRB approval was not sought for this analysis as all data were deidentified and there was no possibility of linking enrollment data to individual patients.

## CONFLICT OF INTEREST

The authors have no conflicts of interest to disclose.

## AUTHOR CONTRIBUTION

Michael Roth: Conceptualization, formal analysis, and writing–review, and editing. Archie Bleyer: Conceptualization and writing–review and editing. Melissa Beauchemin: Interpretation of data, writing, reviewing, and editing. Justine Kahn‐ Interpretation of data, writing, reviewing, and editing.

## Data Availability

Data was obtained from the NCI Cancer Therapy Evaluation Program (CTEP). Requests to access the data can be made directly to CTEP staff.
